# Directions for Using Bromine

**Published:** 1863-04

**Authors:** M. Goldsmith


### CHICAGO MEDICAL JOURNAL.

VOL. VI.] APRIL, 1863. [No. 4.

### ORIGINAL COMMUNICATIONS.

### DIRECTIONS FOR USING BROMINE.

By Dr. M. GOLDSMITH.

U. S. Sanitary Commission, Louisville, Feb. 27, 1863.

The following formula is that used in the preparation of the accompanying preparation of Bromine:

Water, ʒ 10; Bromide of Potassium, ℥ 2½; dissolve the Bromide of Potassium, and add Bromine ℥ 10.

DIRECTIONS FOR USE.

*First*—For Fumigation: Place one ounce of the fluid in a glass tumbler, and place the vessel, one or more, in, according to dimensions of the apartment, at different points, in number sufficient to secure the constant presence of the odor of Bromine in the ward.

It must be borne in mind, that if the vapor of Bromine comes in contact with the vapor of water, Hydro-bromic Acid is formed, and that, therefore, when there is much of the vapor of water disengaged in the apartment, the quantity of the vapor of Bromine must be correspondingly increased.

*Second*—As a Topical application, the Bromine is used in *vapor*, or in *substance*.

For the topical application of the *vapor*, a piece of dry lint is to be placed over the diseased part. Over this is to be placed another piece of lint, moistened in the solution of Bromine; over this, another piece of lint spread with simple cerate, and the whole to be covered with oiled silk and bandage, so arranged, as to retain the vapor in contact with the diseased surface as long as possible. The Bromine is to be removed as frequently as it is exhausted by evaporation.

As a topical application in *substance*, in Hospital Gangrene, Diphtheria, Gangrene of the tongue, *et id omne genus*.

The parts are first to be dried by the application of Charpie. And if the sloughs are thick, they should be trimmed out with scissors and forceps, as much as possible; for the thinner the slough, the more effective is the remedy. The parts being again dried, the solution is applied by means of a mop, or with a pointed stick of wood, in quantity sufficient to saturate the slough. If the sloughs undermine the skin, or dip down into inter-muscular spaces, the Bromine must be inserted by a glass syringe, or with the pointed stick. If the application has been effectual, all odor from the diseased surface ceases, and the sloughs become somewhat hardened. The remedy should be re-applied every second hour, as long as any odor of putrefaction is present, or the sloughs appear to be diffluent. It is not always necessary, especially when the slough's covering are diffluent and thin, to use the solution in full strength. As the disease subsides, the strength of the solution may be diminished by the addition of water.

The points, in the application of the Bromine, to especially attend, are: 1st. That it should be applied in strength and frequency sufficient for the impregnation of the whole of the sloughs. And, 2nd, to this end the application should be made by the surgeon himself, and never entrusted to a nurse.

After the topical application of the solution, the part, when so situated as to make it practicable, should be subjected to the influence of the vapor—see page 1.

Surgeons will do well to bear in mind, that Bromine is a new remedy for the purposes above indicated. The accompanying directions are those followed in the hospitals at this place. It may be found advisable to modify them, as experience accumulates with reference to its use. It is, therefore, earnestly recommended that the subject be studied diligently; that the effects of the remedy be carefully watched; and the application varied, as new facts are developed in the course of its application.

---

[REMARKS.—We have long been in the practice of using the vapor of Iodine in indolent ulcers, and Tr. Iodine in sloughing wounds, in the manner here recommended for Bromine. Dr. Mitchell, in his work on *materia medica*, recommended the vapor of Iodine as a disinfectant, and stated that its presence prevented the communication of contagious diseases.

Iodine, Bromine and Chlorine have similar chemical relations to animal substances and destroy all poisons of the putrefactive class, and some whose classification is doubtful as that of serpents. The advantage of Iodine and Bromine is that they are less active than Chlorine, and can be more thoroughly applied to wounds. Experience must determine which is to be preferred. D. B.]

